# Saffold Virus, a Human Cardiovirus, and Risk of Persistent Islet Autoantibodies in the Longitudinal Birth Cohort Study MIDIA

**DOI:** 10.1371/journal.pone.0136849

**Published:** 2015-08-28

**Authors:** German Tapia, Håkon Bøås, Eric J. de Muinck, Ondrej Cinek, Lars C. Stene, Peter A. Torjesen, Trond Rasmussen, Kjersti S. Rønningen

**Affiliations:** 1 Department of Genes and Environment, Division of Epidemiology, Norwegian Institute of Public Health, Oslo, Norway; 2 Center for Ecological and Evolutionary Synthesis (CEES), Department of Biosciences, University of Oslo, Oslo, Norway; 3 Department of Paediatrics, 2nd Faculty of Medicine, Charles University in Prague and University Hospital Motol, Prague, Czech Republic; 4 Department of Chronic Diseases, Division of Epidemiology, Norwegian Institute of Public Health, Oslo, Norway; 5 Hormone Laboratory, Department of Medical Biochemistry, Oslo University Hospital, Oslo, Norway; 6 Department of IT and e-health, Division of Institute Resources, Norwegian Institute of Public Health, Oslo, Norway; 7 Department of Pediatric Research, Oslo University Hospital, Oslo, Norway; Kliniken der Stadt Köln gGmbH, GERMANY

## Abstract

The aim of this study was to describe the frequency and distribution of Saffold virus in longitudinal stool samples from children, and test for association with development of persistent autoantibodies predictive of type 1 diabetes. A cohort of Norwegian children carrying the HLA genotype associated with highest risk of type 1 diabetes (“DR4-DQ8/DR3-DQ2”) was followed with monthly stool samples from 3 to 35 months of age. Blood samples were tested for autoantibodies to insulin, glutamic acid decarboxylase_65_ and Islet Antigen-2. 2077 stool samples from 27 children with ≥2 repeatedly positive islet autoantibodies (cases), and 53 matched controls were analysed for Saffold virus genomic RNA by semi-quantitative real-time reverse transcriptase PCR. Saffold virus was found in 53 of 2077 (2.6%) samples, with similar proportions between cases (2.5%) and controls (2.6%). The probability of being infected by 3 years of age was 28% (95% CI 0.18–0.40). Viral quantities ranged from <1 to almost 10^5^ copies/μl. Estimated odds ratio between islet autoimmunity and infection episodes prior to seroconversion was 1.98 (95% CI: 0.57–6.91, p = 0.29). Saffold virus had no statistically significant association with islet autoimmunity.

## Introduction

Type 1 diabetes is an autoimmune disorder, believed to result from interactions between a susceptible genetic background and environmental factors. Identification and confirmation of environmental triggers remains a formidable challenge [1,2].

Several viruses are suspected to be involved in the development of type 1 diabetes, in particular picornaviruses [3–7]. The genus *Cardiovirus* (family *picornaviridae*) includes *encephalomyocarditis virus* (ECMV) and *Theilovirus* species. Certain strains of EMCV are highly diabetogenic in mice [8,9], but lack a clear human counterpart [8]. Until recently it was unclear whether this genus included any human pathogens, although some, such as Theilovirus Vilyuisk virus [10] have been suspected. The first clear human cardiovirus, Saffold virus (SAFV), was discovered in 2007 [11]. Subsequently, SAFV has been found in stool [12–19], respiratory [20,21], sewage [22], cerebrospinal fluid, blood and myocardium samples [15], and seems to infect young children [23]. The distribution and associated symptoms of SAFV are still not well described, but SAFV has been reported in both asymptomatic and symptomatic infections, as is also the case for other human picornaviruses such as enteroviruses and parechoviruses [24,25]. Given the associated symptoms and diabetogenic potential of cardioviruses in rodents, and of related viruses in the picornaviridae family in humans, it is of interest to study the potential prospective association of SAFV with reported symptoms of disease and with development of islet autoimmunity and type 1 diabetes.

We aimed to describe the frequency and distribution of SAFV in longitudinal stool samples from children, and test whether SAFV is associated self-reported symptoms of disease or with the development of persistent autoantibodies predictive of type 1 diabetes.

## Materials and Methods

### Subjects and study design

The children included in this study participate in ‘Environmental Triggers of Type 1 Diabetes: The MIDIA study’, which is described in detail by Stene et al. [26]. Briefly, 46,939 Norwegian new-borns were screened for the HLA-DQ-DR genotype conferring the highest risk of type 1 diabetes (HLA-DRB1*04:01-DQA1*03-DQB1*03:02/DRB1*03-DQA1*05-DQB1*02), and 911 new-borns carrying this high risk HLA genotype were recruited for further follow up (3 of these families later withdrew and requested their data to be deleted). A flow-chart of the recruitment is shown in S1 Fig Blood samples were taken and tested for type 1 diabetes-associated autoantibodies at 3, 6, 9 and 12 months of age, and every 12 months thereafter. In the case of an autoantibody positive sample, sampling frequency was increased to every 3–6 months after 12 months of age. Monthly stool samples were collected between 3 to 35 months of age. Information on symptoms of infection (coughing and sneezing, diarrhoea, vomiting, or fever) was recorded in questionnaires at the same ages as the regular blood samples by the parents. At least one of the parents of children included in the MIDIA study had Norwegian or other European origin (the majority had two Norwegian parents). Written parental consent was obtained. The study was approved by the Regional Committee for Medical Research Ethics (Office for Human Research Protections IRB name ‘Regional Med Resch Ethics Comm South IRB #2—South-East A’, IRB00001871) and the Norwegian Data Protection Authority.

#### Islet autoantibodies and case-definition

Blood plasma was assayed for autoantibodies against glutamic acid decarboxylase 65 (GAD), Islet antigen 2 (IA2) or insulin (IAA) as previously described [26]. The end-point was defined as at least two different autoantibodies in at least two consecutive blood samples, or one autoantibody and diagnosis of type 1 diabetes. In this study, 27 children (10 boys and 17 girls) reached the endpoint by 2008, and were assigned as case children. In subjects who first tested positive for one autoantibody, and then developed a second autoantibody at a later stage, the time of occurrence of the first autoantibody was regarded as the first time of positivity. For each case child, two children were randomly selected from the cohort, matched for duration of follow-up (at least up to the time of endpoint), date of birth (±1 month, tolerating up to 3 months in a few cases where necessary), and county of residence. Samples were collected during 2001–2008. Briefly, 80 children were included in the analysis (born between 2001 and 2006) with 2077 stool samples tested for SAFV. Detailed characteristics of the study participants are given in Table 1.

**Table 1:** Characteristics of the participants in this study.

		Case children	Control children	Total
Number of children		27	53	80
Sex (males/females)		10/17	30/23	40/40
Number of other children in the household	0	5	16	21
	1	9	27	36
	≥2	13	10	23
First degree relatives with type 1 diabetes		10/27	3/53	13/80
Median age (range) at last stool sample^a^		30.2 (9.1–35.8)	35.0 (12.5–37.5)	33.0 (9.1–37.5)
Median age (range) of first autoantibody-positive sample^a^		12.1 (5.9–37.4)	-	-
Median age (range)^a^ ^,^ ^b^		42.4 (9.2–83.8)	38.8 (12.4–84.3)	

^a^ in months

^b^ at the time when all the 27 cases were ascertained by the end of 2008.

### Saffold virus assays

The processing and extraction of stool samples is described in detail earlier [27,28]. Briefly, the samples were received by postal service, diluted and centrifuged. The supernatants were frozen at -80°C until co-purification of RNA and DNA. To develop and test the PCR method and as the positive control in the SAFV screening we used plasmids pSAF404 and delPTH5, described in detail in [29], kindly provided by Drs. Toshiki Himeda and Yoshiro Ohara of Kanazawa Medical University School of Medicine, Japan. The real-time RT-PCR was tested using a dilution of plasmid RNA ranging from 10^−1^ to 10^6^ copies/μl; the RT-PCR consistently detected 1 copy/μl. Plasmid RNA was synthesised by use of the RiboMAX Large Scale RNA Production Systems (Promega, Madison, WI).

To test for the presence of SAFV, an in-house one-step real-time RT-PCR was developed targeting the 5´ UTR region of SAFV. Oligonucleotide sequences and their concentrations in the reactions are given in Table 2. All tests were performed in duplicate using the QuantiTect Probe RT-PCR Kit (Qiagen, Hilden, Germany) and carried out on an Applied Biosystems 7300 Real Time PCR system (Applied Biosystems, Foster City, CA) with the following thermal profile: 30 minutes at 50°C, 15 minutes at 95°C followed by 45 cycles of 15 seconds at 94°C and 1 minute at 60°C. To quantify the amounts of virus present a standard curve ranging from 10^0^ to 10^5^ copies/μl was run alongside the samples, using 2μl of plasmid RNA as template. The viral quantity was set to the mean quantity of the duplicates. No cross-reactivity with human entero- and human parechoviruses was indicated in the PCR by testing several samples known to be positive for these viruses.

**Table 2:** Primers and Probes used in the Study.

	Sequence 5´-3´	Position^a^	Concentration
SAFV-detection^b^			
SAFV_692F	ATGCCGGAAACGGTGAAGA	662	300 nM
SAFV_827R	ACCGCTCACAGCAGTGGATC	817	900 nM
SAFV_743P_MGB^c^	FAM-TCGAAACAGCTGTAGCGAC	713	150 nM
SAFV genotyping			
SAFV_VP1_FC	AYAATGCTGARAAAGGMAARG	2977	500 nM
SAFV_VP1_RG	CCRGGAATTTCATATTGRCA	3904	500 nM

^a^ position given when aligning to NCBI Reference Sequence: NC_009448.2

^b^ F: forward primer; R: reverse primer; P: Probe.

^c^ Minor Groove Binder.

To get information on SAFV genotypes, a PCR targeting the VP1 region was performed on the positive samples using primers described in Table 2. The PCR product was sequenced using standard Sanger sequencing on an ABI 3730 DNA Analyzer (Applied Biosystems, Foster City, CA). A complete protocol is available from the authors upon request.

### Data analysis

A detailed description of analysis methods is given in the S1 File. Briefly, the association of SAFV with islet autoimmunity (and other variables) was primarily analysed using a mixed effects logistic regression model with SAFV infections as the dependent variable. The primary analysis involved only samples collected up to islet autoimmunity for the cases and the corresponding age in the matched controls, but pre-planned sub-group analyses included other time widows such as samples collected to the date of the last autoantibody negative blood sample. To adjust for potential confounders, variables such as sex, first degree relatives with type 1 diabetes, number of siblings in the household and breastfeeding were added as independent variables to the model, excluding missing observations. A midpoint rooted phylogenetic tree was constructed using the obtained VP1 sequences together with other sequences from GenBank. The robustness of the estimated phylogenetic three was determined by bootstrapping with 1000 replicates.

## Results

### SAFV positive stool samples by sex, age and season

The total proportion of SAFV positive samples was 53 of 2077 samples (2.6%). There was no statistically significant difference in the proportion of SAFV positive samples between the sexes (1.65% males, 3.44% females). Overall there were 28 infection episodes (defined as a positive sample, followed by any number of consecutive positive samples) during follow-up.

SAFV positivity was elevated around 12–28 months of age with a peak at 18–21 months followed by an apparent drop (S2 Fig), although the association with age was not significant (p = 0.1). The proportions of positive samples were highest during the winter months (S3 Fig), but this observation was not statistically significant.

### Patterns of SAFV infection in individuals during follow-up

**Fig 1:**
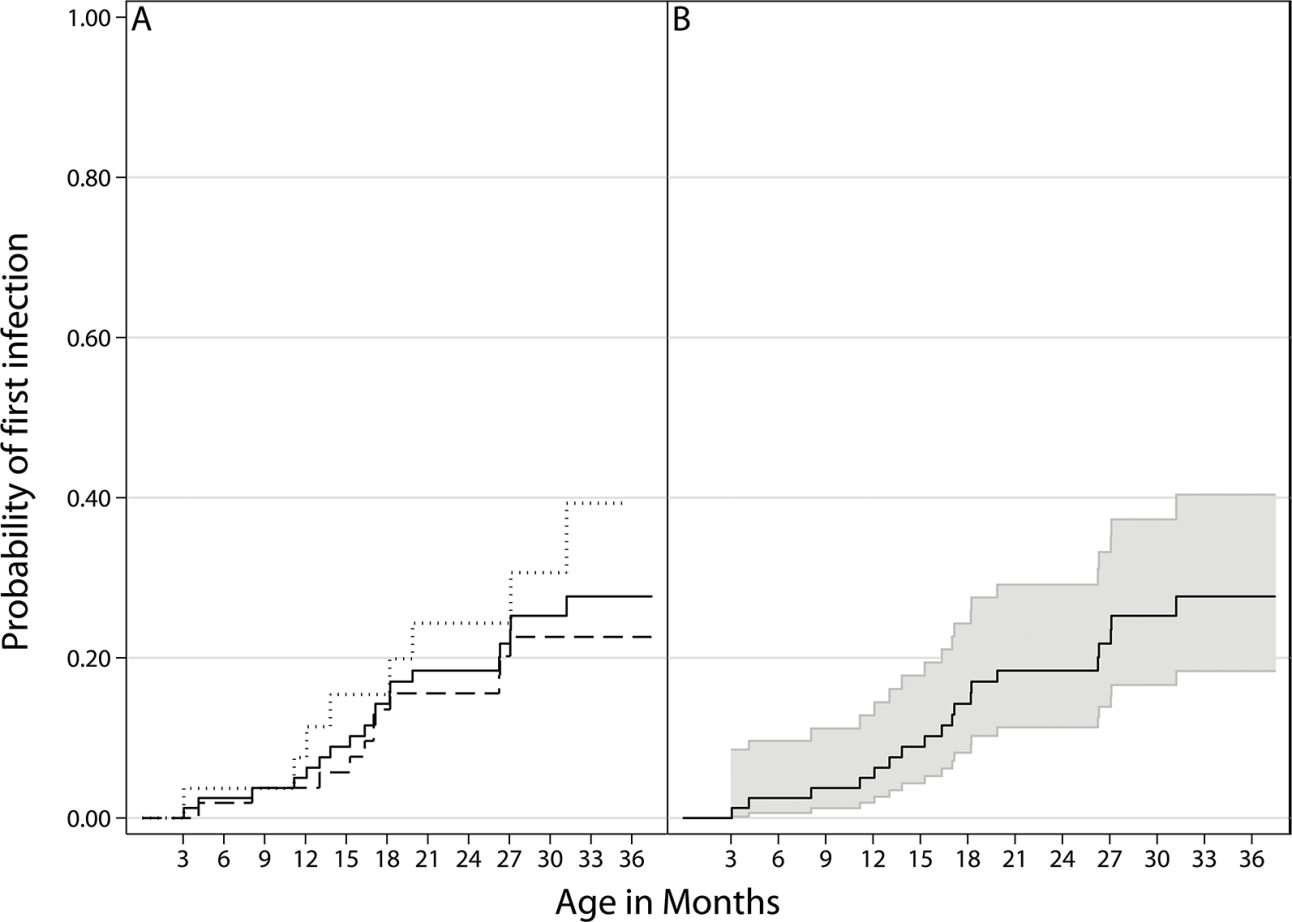
Probability of having the first infection with Saffold virus during follow-up. Probability is calculated as 1 minus the Kaplan-Meier estimate. 2A: All children (solid line), case children (dotted line) and control children (dashed line). 2B: All children (solid line) with 95% confidence interval bands. There is no statistical difference between the case and control children.

Of the 80 children followed, 19 (24%) tested positive for SAFV at least once. The probability of having a SAFV-positive stool sample before 2 years of age was 18% (95% CI 11–29) (Fig 1). Thirteen children had multiple positive samples, with the maximum being seven positive samples in each of two children. Infections often started with a high quantity that dropped over time (S4 Fig). The infection episodes lasted from 1 to 4 consecutive monthly samples, with a median infection length of 2 months for the first SAFV infection. Viral quantities ranged from less than one to 96417 copies/μl, with a median of 164 copies/μl (S1 Table).

### Saffold virus and symptoms of infectious disease

Run separately, symptoms of the common cold, diarrhoea or fever were not significantly associated with the presence of SAFV RNA in faeces (results not shown). However, when including all of these symptoms as independent variables in the model, there was a tendency for SAFV positive samples to occur at the same time as symptoms of the common cold were reported by the parents (uncorrected OR 2.43, 95% CI 1.00–5.90, p = 0.051). Diarrhoea or fever remained not significantly associated with the presence of SAFV RNA in faeces. Due to missing observation only 1479, 1629 and 1561 were included in the analysis of common cold, diarrhoea or fever respectively. In the combined model 1371 observations were included in the analysis.

### Saffold virus and with type 1 diabetes- associated islet autoimmunity

The observed frequency of SAFV infections was slightly higher among case children than among matched control children, but the difference was not statistically significant (Table 3). Adjusting for sex, number of siblings in the household, breastfeeding or first degree relatives with type 1 diabetes did not have a major impact on the conclusions (data not shown). Due to missing observations only 1911 observations were included in the adjusted analysis.

**Table 3:** Saffold virus infection episodes in the case and control children.

	Case children (%)	Control children (%)	OR, 95% CI
Whole observation period	10/644 (1.55%)	18/1433(1.26%)	1.27, 0.58–2.78
After IA	5/284 (1.76%)	13/724 (1.80%)	1.06, 0.36–3.08
Before IA	5/360 (1.39%)	5/709 (0.71%)	1.98, 0.57–6.91

The number of infection episodes and total samples, with the percent in parentheses, and the results from the mixed effects logistic regression are given for case and control children at different time periods. IA: islet autoimmunity; OR: Odds Ratio; 95% CI: 95% Confidence Interval.

Conditional logistic regression analysis gave similar results (odds ratio for ≥1 vs 0 infections: 2.25, 95% CI: 0.53–9.63, p = 0.27). There was no significant difference after restriction to specific predefined time periods prior to the appearance of autoantibodies (S2 Table), nor when only using samples taken before and concurrent with the last autoantibody negative blood sample. Using infections occurring together with reported symptoms, infections lasting ≥2 months or positive samples in lieu of infection episodes in the analysis gave similar results (data not shown). Neither were there any significant differences between case children and control children in the viral quantities (median 174 vs. 164 for cases and controls, respectively) or length of infection (median 1.5 vs. 1 for all SAFV infections in case and control children, respectively).

We also tested whether our results were influenced by the choice of positivity threshold, but the lack of significant association remained also after increasing this to 10, 1000 or 10000 copies/μl in the statistical analysis (data not shown). Finally, we ran the analysis using only infections prior to 6 months of age, and prior to 12 months of age, again with no significant results (data not shown).

### Sequencing results

Partial VP1 sequences were obtained from 23 of the 53 positive samples. In general, samples with low viral quantities were unsuccessful. There was 16 SAFV-2 and 7 SAFV-3 positive samples; other genotypes were not found in our study. Two children were infected with both genotypes at different times (S4 Fig), and re-infection with the same genotype at a later time was also observed.

**Fig 2:**
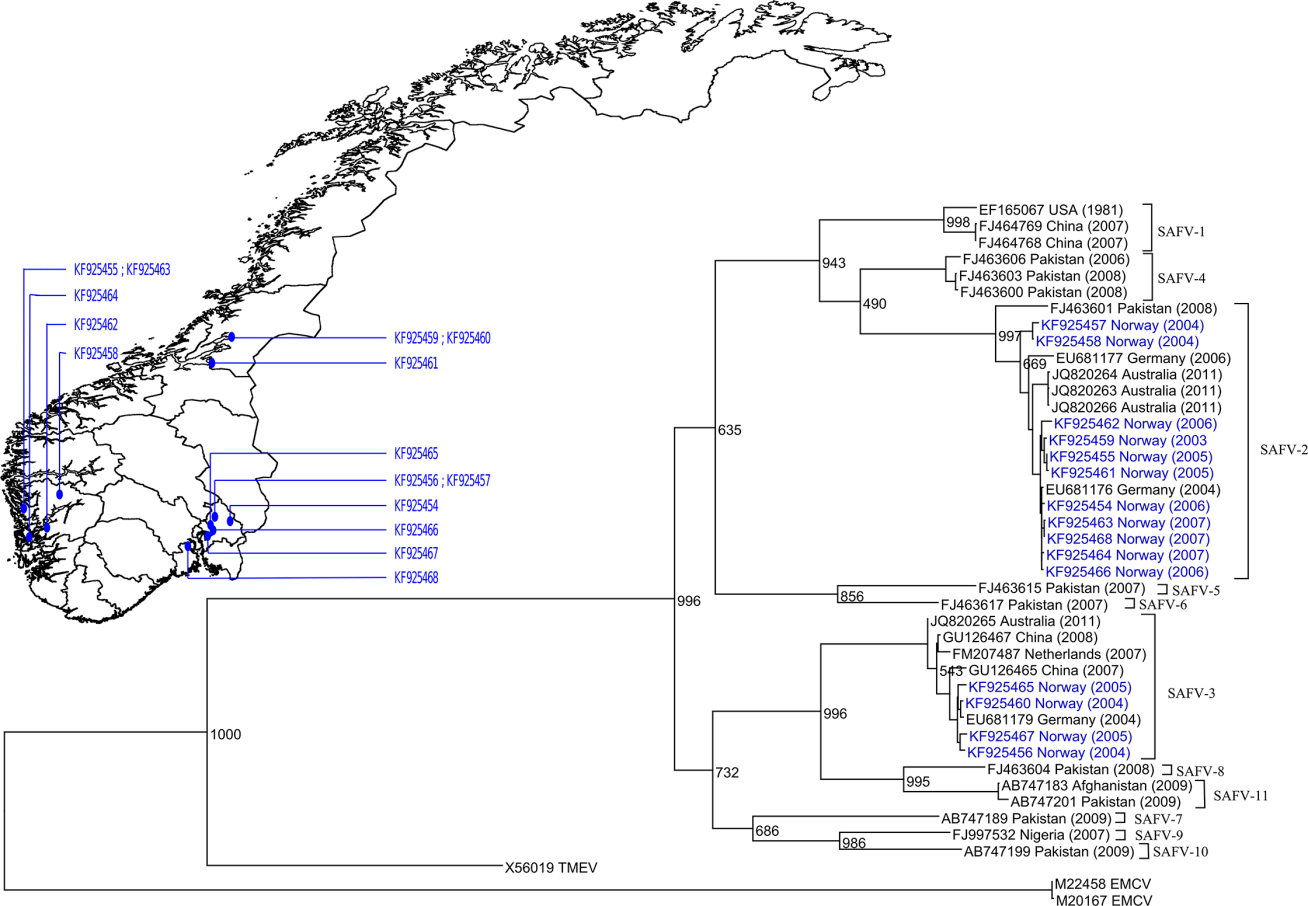
Infections and GenBank sequences in relation to Norwegian municipalities. Phylogenetic tree of the Saffold virus genotypes and geographical distribution of sequenced samples. Strains from the current study are colored blue. Sequences are denoted with GenBank accesion number, then the country of origin and year of sampling in parentheses. The tree is rooted by using midpoint rooting Scale bars represent 0.1 nucleotide substitutions per site. Only one sequence per infection episode is presented in the phylogenetic tree as the dominating sequence seemed stable and there were no evident mutations in the VP1 sequence during an infection (results not shown), supporting the assumption that consecutive positive samples were part of the same infectious episode.

A phylogenetic tree of the sequences, and their geographical distribution, is presented in Fig 2. Generally, the strains observed clustered together in regards to geography and time period, showing that different strains were circulating at different times/locations in Norway. The clusters should not be taken as local outbreaks, as the areas with most positive samples are areas of high population density in Norway, which contribute most of the participants in the cohort.

## Discussion

In this first large-scale, longitudinal study of SAFV and type 1 diabetes-associated islet autoimmunity, we did not find any significant association. We further described the frequency of SAFV by age, season and infectious symptoms in one of the largest, longitudinal study of otherwise healthy children published to date.

We are only aware of a single previous study that attempted to investigate the relation between SAFV and islet autoimmunity [30], but with only 49 stool samples assayed and none observed to be SAFV-positive, that study was clearly underpowered. Similar to our study the participants had a high genetic risk of type 1 diabetes, and the results can not necessarily be generalised to the general population. However our results will be relevant for a major fraction of future type 1 diabetes cases [31]. While seroepidemiological studies suggest that SAFV is ubiquitous [23,32], we observed infections in about a quarter of our children during follow-up and the proportion of SAFV-positive stool samples was relatively low. Among more than 2000 stool samples tested in our study, 2.6% were positive, which seems to be in line with most other available studies of SAFV occurrence [12–14,16,17,33]. This is considerably lower than what we have previously found for enterovirus and parechovirus in the same samples [6,27]. This relatively low frequency of SAFV positivity suggest that extremely large scale studies are necessary to rule out possible associations of modest to small magnitude of SAFV with islet autoimmunity, type 1 diabetes or symptoms of infections. Although it is possible that diabetogenic SAFV strains exist, similar to the EMCV-D strain in mice [8,9], these would be expected to be the cause of a relatively small proportion of type 1 diabetes cases.

Most available studies of SAFV occurrence based on PCR detection were conducted on patients with symptoms of infectious disease, and included only one or a few samples from each subject during a limited time period. Few previous publications have reported SAFV occurrence in population based studies. A recent Danish study found 2.8% of stools to be positive, consistent with our observation [14]. In another recent Finnish study, SAFV was tested in 160 stool and nasal swab samples collected at the time of infectious disease symptoms from 45 children, resulting in only one SAFV positive stool sample [18]. Other smaller studies of symptomatic children have reported from 1.1% to 3.2% positive samples [12,13,16,17,33], whereas a study of German children reported 7.8% positive stool samples [12] and two studies of Pakistani and Afghani children reported ~10% positive stool samples [19,34]. Some random variation is to be expected with the small number of observed positive samples and total samples in these studies, and there is also variation in age and clinical characteristics which make direct comparisons difficult. While there may be many other factors influencing the frequency of SAFV infection, it seems at current that there are no large differences in observed frequency between studies of symptomatic children and population based studies, which is also consistent with the lack of association with common infectious disease symptoms (fever, diarrhoea, vomiting or common cold symptoms) in our study. It is worth noting that SAFV have been found in invasive infections [15]. It seems likely that SAFV may act similarly to other picornaviruses with generally asymptomatic infections that may cause various serious conditions in a small fraction of cases.

We observed some variation by age and season, and although this was not statistically significant, we would expect that the true frequency of SAFV varies to some extent with these variables. When comparing SAFV frequency among studies, we therefore believe that both age and season are important to consider, but perhaps most importantly the frequency and duration of longitudinal sample collection. The fact that we observed on average two consecutive positive monthly stool samples suggests that monthly samples should be sufficient to detect most infections, although we cannot rule out the possibility that infections with faecal shedding for less than a month were missed.

Detection of SAFV in stool samples with PCR methods should be highly sensitive, but it is important to consider the sensitivity of the PCR and the specificity of the primers. For instance, the previously mentioned studies that report higher proportion of positive samples used nested PCR, targeting the 2C region of SAFV [34] and 5-UTR [12,19], while ours and others mentioned targeted the 5’ UTR and did not use nested PCR. Of necessity, PCR primers can only be designed based on the currently known SAFV sequences. While we do not expect this to be a major problem because the 5’ UTR tend to be well conserved, we cannot rule out the possibility that there are other SAFV with sequences for which our primers have limited sensitivity.

While there seems to be geographical differences in the proportion of SAFV positive samples when comparing the studies from Germany [12], Pakistan and Afghanistan[19,34] to those from Denmark[14] and the current study from Norway, these differences could be due to the methods used (nested vs. non-nested PCR), or due to the fact that the studies from Germany, Pakistan and Afghanistan included older children (≤12 and ≤15 years of age, respectively) while the Scandinavian studies are in younger children (≤3 years of age). It is worth noting that seroepidemiological results are similar in several countries (Netherlands, Finland, Cameroon, Indonesia), with >75% having antibodies against SAFV by 24 months of age [23,32]. This speaks against geographical or socioeconomic differences in frequency of SAFV infections in young children. The apparent discrepancy between the SAFV-frequency in PCR based studies as ours and seroepidemiological studies may be due to a variety of factors. On one hand, short duration and infrequent sampling of stool samples used for PCR testing might lead to underreporting. On the other hand, cross-reactivity and limited specificity of the antibody assays, or SAFV infections outside the gut, might be the reason for the higher reported cumulative incidence in seroepidemiological studies.

The SAFV genotypes found in this study (SAFV-2 and -3) seem to be the more commonly reported strains also in other studies [12,14,16]. Our findings are similar to what was found during similar time periods in the Netherlands [32] and in Denmark [14], indicating that these genotypes are the most commonly found in Western Europe. Unfortunately, the number of samples positive for each specific SAFV genotype was too low for any meaningful interpretation of associations with age, season, symptoms or islet autoimmunity in our study.

## Conclusions

In this first large-scale study of SAFV and islet autoimmunity, we found 2.6% positives among more than 2000 monthly stool samples from children at genetic risk of type 1 diabetes who were followed from 3 months of age. While we observed a slightly higher frequency among those who later developed islet autoimmunity, the association was not significant. We found no significant associations between SAFV infections and reported symptoms of infection, which should be kept in mind when evaluating a positive finding in stool. Based on our observations, we can conclude that extremely large studies are necessary to exclude potential associations of modest magnitude, particularly if investigating specific SAFV genotypes.

## Supporting Information

S1 Fig Recruitment flowchart. (PDF)

S2 Fig Saffold virus positive samples by age. Observed proportion of samples in age-groups (≤6, 6–9, 9–12, 12–15, 15–18, 18–21, 21–24, 24–27, 27–30, 30–33 and ≥33 months) with confidence intervals based on binomial proportions (ignoring potential clustering within subjects and matched sets). Smoothed line based on prediction from logistic regression model with age (at time of sample collection) and age squared.
(TIF)

S3 Fig Seasonality of the positive samples. Observed proportion of positive samples per calendar month with confidence intervals based on binomial proportions (ignoring potential clustering within subjects and matched sets). Smoothed line based on prediction from logistic regression model with month (at time of sample collection) and month squared.
(TIF)

S4 Fig Data from matched sets of all individuals with at least one positive SAFV-sample. Samples from the matched groups where at least one child had a positive sample. Infections in four selected children are shown in more detail; detected genotype (where known) is indicated next to each infection episode. ○: negative stool sample, ●: positive sample, ×: time of first autoantibody positive blood sample in a case child.
(TIF)

S1 File Supporting information on data analysis. (DOCX)

S1 Table Viral quantities and duration of infection. (DOCX)

S2 Table Analysis at different time periods before development of islet autoimmunity. (DOCX)
