# Supplementary material for: Saffold Virus, a Human Cardiovirus, and Risk of Persistent Islet Autoantibodies in the Longitudinal Birth Cohort Study MIDIA
Source: PLoS One. 2015 Aug 28;10(8):e0136849. doi: 10.1371/journal.pone.0136849 (PMC4552579; doi:10.1371/journal.pone.0136849)

Supplementary Material:

Supplementary Figure S1: Flow diagram depicting the study participants

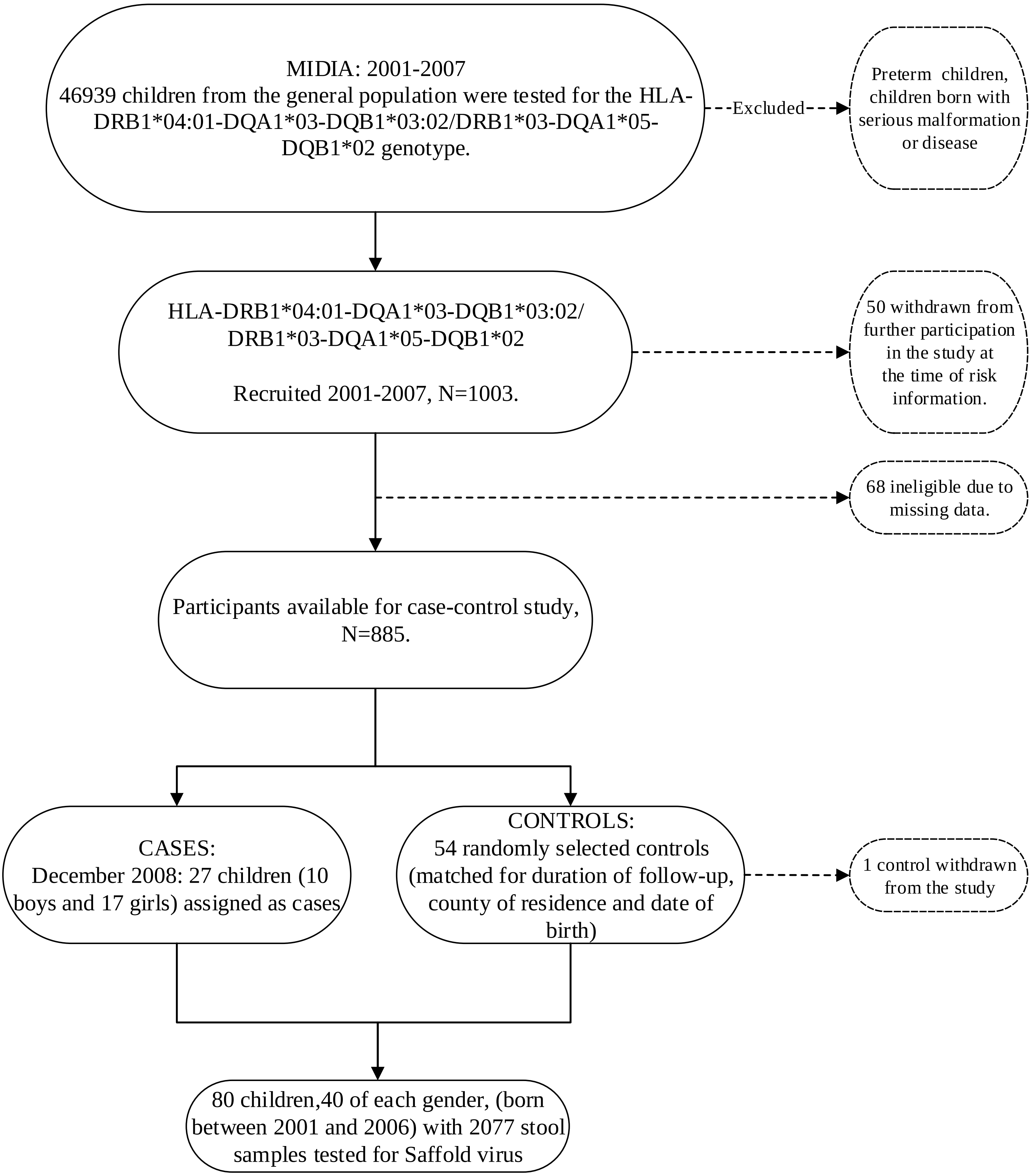

Supplement: S1 Fig — (PDF) [file pone.0136849.s001.pdf]
