# Supplementary material for: Saffold Virus, a Human Cardiovirus, and Risk of Persistent Islet Autoantibodies in the Longitudinal Birth Cohort Study MIDIA
Source: PLoS One. 2015 Aug 28;10(8):e0136849. doi: 10.1371/journal.pone.0136849 (PMC4552579; doi:10.1371/journal.pone.0136849)
Supplement: S1 File — (DOCX) [file pone.0136849.s005.docx]

**Supporting information on data analysis**

The association of Saffold virus (SAFV) with islet autoimmunity (and other variables) was primarily analysed using a mixed effects logistic regression model with SAFV infection (using only the first SAFV positive sample among series of two or more consecutively positive samples, assuming they were part of the same infectious episode) as the dependent variable and islet autoimmunity case/control status as the independent variable. To account for the repeated measurements within subjects and the matched design, we specified random intercepts for each individual and matching group (using the command xtmelogit procedure with default settings in Stata, version 13). Additionally, the analyses were run using all SAFV positive samples. The estimated odds ratio (with 95% confidence interval) from this model is interpreted as the odds that a fecal sample is positive for SAFV RNA given that it came from a child who later developed islet autoimmunity, relative to the odds that a sample is SAFV RNA positive given that it came from a matched control child. In addition, we supplemented the above analysis with a traditional conditional logistic regression analysis accounting for the matching, and using zero vs. >=1 infection during follow-up as the independent variable. Onset of islet autoimmunity was defined as the time of the first autoantibody positive blood sample. The primary analysis involved only stool samples collected up to onset of islet autoimmunity for the cases and the corresponding age in the matched controls. Pre-planned sub-group analyses (using the mixed effects logistic regression model) were done for time windows of 3, 6, 9 and 12 months prior to islet autoimmunity in cases (and corresponding ages in matched controls), samples collected prior t o the last autoantibody-negative blood sample, samples collected prior to one year of age, and samples collected after islet autoimmunity. The probability of testing positive for the first time during follow-up (the failure function) was estimated using the Kaplan-Meier method (calculated by 1 minus the Kaplan-Meier estimate). The association of SAFV positive samples with season and age was tested by stratified analysis and logistic regression models with polynomial terms for age (age plus age square) and season (week 1-52 of the year plus square and cubic terms for week). Significance of age and season were tested using the likelihood ratio test for models with and without the relevant variables. Differences in case and control children in the distribution of viral quantities and length of infection were tested using a nonparametric K-sample test.

A midpoint rooted phylogenetic tree was constructed using the obtained VP1 sequences together with a representative collection other sequences available from GenBank. The sequences were aligned using CLUSTALW [1]. The alignments were manually curated and phylogenetic analysis performed using the maximum likelihood phylogeny in the PhyML 3.0 software package [2], on the basis of the best-ﬁt TPM2uf+I+Γ nucleotide model as determined by jModelTest [3]. The robustness of the estimated phylogenetic three was determined by bootstrapping with 1000 replicates.

**References**

1. Larkin MA, Blackshields G, Brown NP, Chenna R, McGettigan PA, McWilliam H, Valentin F, Wallace IM, Wilm A, Lopez R, Thompson JD, Gibson TJ, Higgins DG: Clustal W and Clustal X version 2.0. Bioinformatics 2007;23:2947-2948

2. Guindon S, Dufayard JF, Lefort V, Anisimova M, Hordijk W, Gascuel O: New algorithms and methods to estimate maximum-likelihood phylogenies: assessing the performance of PhyML 3.0. Systematic biology 2010;59:307-321

3. Darriba D, Taboada GL, Doallo R, Posada D: jModelTest 2: more models, new heuristics and parallel computing. Nature methods 2012;9:772
