# Supplementary material for: Saffold Virus, a Human Cardiovirus, and Risk of Persistent Islet Autoantibodies in the Longitudinal Birth Cohort Study MIDIA
Source: PLoS One. 2015 Aug 28;10(8):e0136849. doi: 10.1371/journal.pone.0136849 (PMC4552579; doi:10.1371/journal.pone.0136849)
Supplement: S1 Table — (DOCX) [file pone.0136849.s006.docx]

**Supporting table 1: Viral quantities and duration of infection**

| **Viral quantity/μL of sample (rounded)** | **Number of positive samples (%)** | **Number of positive children^a^** |
| --- | --- | --- |
| 10^5^ | 2/53 (3.8) | 2/19 |
| 10^4^ | 7/53 (13.2) | 5/19 |
| 10^3^ | 15/53 (28.3) | 6/19 |
| 10^2^ | 7/53 (13.2) | 3/19 |
| 10^1^ | 6/53 (11.3) | 1/19 |
| <5 copies pr. μL | 16/53 (30.2) | 2/19 |
| **Duration of infection** |  |  |
| 4 months | 4/28 (14.3) | 4/19 |
| 3 months | 4/28 (14.3) | 2/19 |
| 2 months | 5/28 (17.9) | 5/19 |
| 1 month | 15/28 (53.6) | 8/19 |

^a^ Only the highest viral quantity or longest infection in each child is counted
