# Supplementary material for: Saffold Virus, a Human Cardiovirus, and Risk of Persistent Islet Autoantibodies in the Longitudinal Birth Cohort Study MIDIA
Source: PLoS One. 2015 Aug 28;10(8):e0136849. doi: 10.1371/journal.pone.0136849 (PMC4552579; doi:10.1371/journal.pone.0136849)
Supplement: S2 Table — (DOCX) [file pone.0136849.s007.docx]

**Supporting table 2: Analysis at different time periods before development of islet autoimmunity**

|  | **Case children (%)** | **Control children (%)** | **OR, 95% CI** |
| --- | --- | --- | --- |
| 12 months prior to IA | 3/232 (1.29%) | 4/469 (0.85%) | 1.53, 0.33-6.90 |
| 9 months prior to IA | 3/204 (1.47%) | 4/411 (0.97%) | 1.53, 0.34-6.96 |
| 6 months prior to IA | 3/158 (1.90%) | 2/316 (0.63%) | 3.04, 0.50-18.37 |
| 3 months prior to IA | 1/90 (1.11%) | 0/186 (0%) | n.a |

Number of infection episodes and total number of samples in case and control children at selected time intervals prior to development of islet autoimmunity. The association of SAFV with islet autoimmunity was analysed using a mixed effects logistic regression model with SAFV infections as the dependent variable.

IA: islet autoimmunity; OR: Odds Ratio; 95% CI: 95% Confidence Interval
